# Supplementary material for: Comparative multiomics analysis of cell physiological state after culture in a basket bioreactor
Source: Sci Rep. 2022 Nov 23;12:20161. doi: 10.1038/s41598-022-24687-4 (PMC9686226; doi:10.1038/s41598-022-24687-4)
Supplement: Supplementary file 1 — Supplementary Information 1. [file 41598_2022_24687_MOESM1_ESM.zip › raw data/Proteomic raw data/4.DiffExprAnalysis/EG.vs.CG/EG.vs.CG.heatmap.detail.pdf]

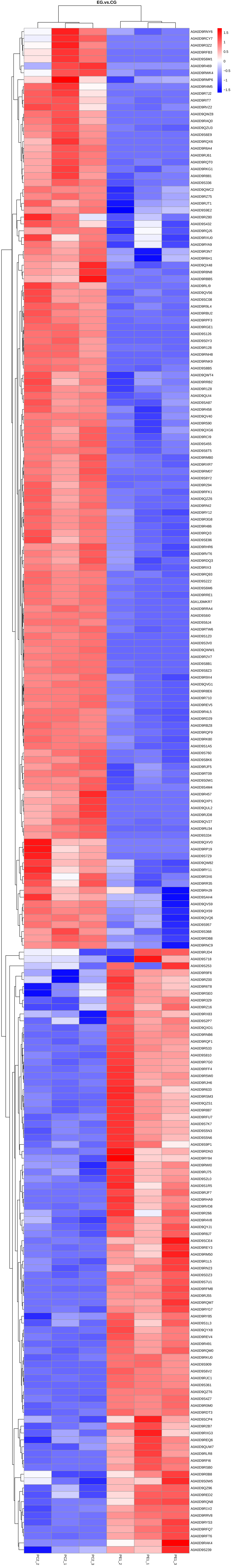
EG.vs.CG
1.5
1
0.5
0
−0.5
−1
−1.5
PC_2
PC_1
PC_3
PE_2
PE_1
PE_3
